# Measurement invariance of HIV-related stigma scales among men who have sex with men (MSM) and non-MSM populations: implications for comparative studies in China

**DOI:** 10.3389/fpsyg.2025.1510034

**Published:** 2025-04-25

**Authors:** Tianyue Mi, Xueying Yang, Guanghua Lan, Shan Qiao, Zhiyong Shen, Yuejiao Zhou, Xiaoming Li

**Affiliations:** ^1^Department of Health Promotion, Education, and Behavior, South Carolina SmartState Center for Healthcare Quality (CHQ), University of South Carolina, Columbia, SC, United States; ^2^Guangxi Center for Disease Control and Prevention, Nanning, China

**Keywords:** measurement invariance, HIV-related stigma, men who have sex with men (MSM), internalized stigma, anticipated stigma, enacted stigma, cross-group comparison

## Abstract

**Background:**

Measurement invariance ensures that scales used in research measure the same constructs across different groups. As HIV-related stigma scales are increasingly used in studies involving men who have sex with men (MSM) and non-MSM populations, it is crucial to evaluate the equivalence of these measures. This study examines the measurement invariance of internalized, anticipated, and enacted HIV-related stigma scales between MSM and non-MSM populations in China.

**Methods:**

Data were derived from two studies: a prospective cohort study with 193 MSM and 579 non-MSM, and a cross-sectional survey of 402 MSM. Participants completed the 8-item internalized, 9-item anticipated, and 16-item enacted HIV-related stigma scales. Confirmatory factor analysis was used to test measurement invariance by progressively adding equality constraints to the models for each stigma dimension.

**Results:**

Partial scalar measurement invariance was achieved for the internalized stigma scale, allowing the intercepts of items 2, 3, and 6 to vary (χ^2^ = 89.32, *df* = 43; CFI = 0.986; TLI = 0.981; RMSEA = 0.043, 95%CI [0.030, 0.056]; SRMR = 0.033), indicating that the zero points of item 2 (“I feel ashamed of having HIV”), item 3 (“Having HIV makes me feel unclean”), and item 6 (“I feel guilty because I have HIV”) were different between MSM and non-MSM. Partial residual measurement invariance was established for the anticipated stigma scale by allowing the residuals of item 2 to vary (χ^2^ = 93.57, *df* = 66; CFI = 0.994; TLI = 0.993; RMSEA = 0.027, 95%CI [0.012, 0.038]; SRMR = 0.022), indicating that the item variance that could not be explained by the factor was different between MSM and non-MSM. For the enacted stigma scale, partial scalar invariance was achieved by allowing the threshold of item 7 to vary (χ^2^ = 314.74, *df* = 219; CFI = 0.987; TLI = 0.986; RMSEA = 0.027, 95%CI [0.020, 0.034]; SRMR = 0.088), indicating that the threshold of item 7 was different between MSM and non-MSM.

**Conclusion:**

The study supports the use of these HIV-related stigma scales for comparing MSM and non-MSM populations, though caution is needed as some items demonstrated partial measurement invariance. These findings provide a foundation for future research and interventions aimed at reducing HIV-related stigma across diverse groups.

## Introduction

HIV-related stigma, recognized as a discrediting and tainting social label (Goffman, 2009), has been extensively documented as a significant obstacle to the physical and psychological well-being, as well as healthcare access, for people living with HIV (PLWH) (Courtenay Quirk et al., 2006; Dowshen et al., 2009). The Health Stigma Framework delineates three dimensions of stigma experienced by PLWH: internalized, anticipated, and enacted stigma, stemming from perceived prejudice, stereotypes, and discrimination (Earnshaw and Chaudoir, 2009; Earnshaw et al., 2013). Internalized stigma entails the acceptance of negative attitudes associated with HIV and their application to oneself, leading to feelings of self-blame, guilt, and worthlessness (Earnshaw and Chaudoir, 2009). Anticipated stigma involves expectations of discrimination, stereotyping, or prejudice from others upon disclosure of HIV status (Earnshaw et al., 2013). Enacted stigma refers to actual experiences of discrimination, stereotyping, or prejudice due to HIV status, either in the past or present (Earnshaw et al., 2013; Center for Disease Control and Prevention, 2021).

These three dimensions of HIV-related stigma are closely interconnected and have been firmly established as detrimental to the physical, psychological, and behavioral health of PLWH (Cole et al., 1997). HIV-related stigma has been associated with hastened disease progression, evidenced by reduced CD4 counts, elevated viral loads, and accelerated onset of AIDS diagnosis in untreated individuals (Cole et al., 1997; Lyons et al., 2020). Psychologically, HIV-related stigma contributes to a range of mental health disorders among PLWH, including depression, anxiety, emotional distress, and thoughts or attempts of suicide (Lee et al., 2002; Kang et al., 2005; Siegel et al., 2005; Gonzalez et al., 2009; Bogart et al., 2010; Carrico, 2010; Capron et al., 2012). Additionally, a systematic review revealed associations between HIV-related stigma and maladaptive health behaviors such as suboptimal adherence to antiretroviral therapy (Sweeney and Vanable, 2016).

Several scales have been developed to measure the three dimensions of HIV-related stigma. One measurement tool of internalized HIV-related stigma is the 8-item scale derived from the “negative self-image” component of the Berger HIV Stigma Scale, validated with a large and diverse sample of PLWH (Berger et al., 2001). Anticipated HIV-related stigma can be evaluated using a 9-item scale based on the Health Stigma Framework, which assesses participants’ expectations of stigma from family members, communities, and healthcare providers (Earnshaw et al., 2013). Enacted HIV-related stigma can be measured using a 16-item checklist derived from the PLWH Stigma Index, focusing on actual experiences of stigmatization due to HIV within the past 6 months (dos Santos et al., 2014).

As the measurement tools for the three dimensions of HIV-related stigma were developed among PLWH rather than specifically among men who have sex with men (MSM) (Berger et al., 2001), measurement invariance for scales of the three types of HIV-related stigma is a prerequisite for comparative studies between MSM and non-MSM populations. Given potential differences in self-image, experiences of discrimination, or perceptions of social norms between non-MSM and MSM (Yan et al., 2019), it is imperative to ensure that assessment tools for measuring HIV-related stigma are tapping into the same underlying construct across MSM groups. Achieving measurement invariance between MSM and non-MSM populations would enable attributing observed group differences in HIV-related stigma to genuine disparities between the groups rather than measurement inconsistencies (Vandenberg and Lance, 2000). Conversely, if measurement invariance is not established, it suggests that the HIV-related stigma scales may not accurately capture the intended construct, potentially leading to misinterpretation of group differences due to inconsistent comprehension of key concepts or measurement variations (Vandenberg and Lance, 2000).

Measurement invariance has not been examined for the internalized, anticipated, and enacted HIV-related stigma scales between MSM and non-MSM populations, and therefore the suitability of utilizing these instruments across diverse subgroups remains undetermined. This study aims to assess measurement invariance for the three HIV-related stigma scales between non-MSM and MSM populations in China through multi-group comparisons within the framework of confirmatory factor analyses.

## Methods

### Study setting and participants

**Figure 1:**
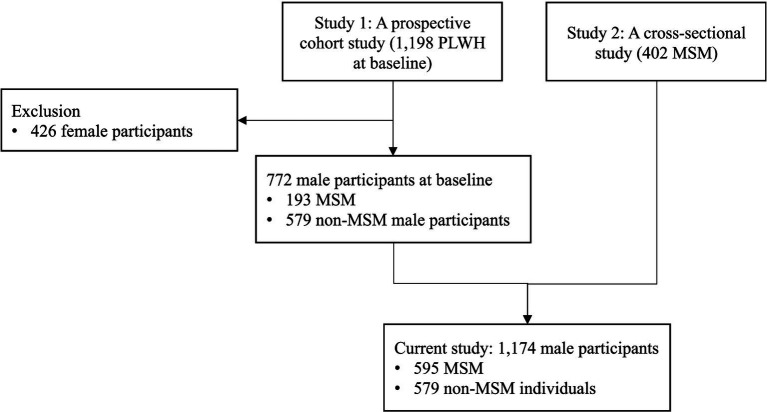
Integration of participants from longitudinal and cross-sectional studies.

As shown in Figure 1, data for this study were derived from a prospective cohort study and a cross-sectional survey conducted in Guangxi, China. The prospective cohort study aimed to investigate the association between HIV-related stigma and clinical outcomes among PLWH, focusing on physical, mental, and behavioral mechanisms. Baseline assessment was carried out between November 2017 and February 2018 in collaboration with the Guangxi Center for Disease Control and Prevention (Guangxi CDC). Six major public hospitals/clinics with the highest volume of HIV patients under care in five cities were selected as study sites. Eligible participants for the cohort study were PLWH aged between 18 and 60 years, with a confirmed HIV diagnosis, and no plans to relocate outside of Guangxi province within the next 12 months. A total of 1,198 PLWH were recruited, of which 64.4% (*n* = 772) were men, including 193 men who have sex with men (MSM) and 579 non-MSM individuals. This subset of men living with HIV (MLWH) were included in the current study.

The cross-sectional survey study aimed to explore the effects of MSM status on HIV-related health outcomes among MSM living with HIV. Data collection took place between August 2020 and May 2021 in collaboration with Guangxi CDC. Four major public hospitals/clinics with the highest cumulative number of MSM living with HIV in three cities were selected as study sites. Eligible participants were men aged 18–60 years, with a confirmed HIV/AIDS diagnosis, and self-reported engagement in sex with men in the last 6 months. MSM who had already participated in the prospective cohort study were excluded, as verified through unique identification numbers in the CDC health record system. After excluding ineligible participants, a total of 402 MSM were included in the current study.

### Assessment instruments

#### Internalized HIV-related stigma scale

Internalized HIV-related stigma was assessed using an 8-item scale derived from the “negative self-image” subscale of the Berger HIV Stigma Scale (Berger et al., 2001). Participants rated each statement on a 4-point scale ranging from 1 (“strongly disagree”) to 4 (“strongly agree”). Sample statements included “I feel I’m not as good as others because I have HIV” and “I feel guilty because I have HIV.” A sum score was calculated from the 8 items, with higher scores indicating higher levels of internalized HIV stigma. The scale demonstrated excellent internal consistency (Cronbach’s *α* = 0.95).

#### Anticipated HIV-related stigma

Anticipated HIV-related stigma was assessed using a 9-item scale based on the Health Stigma Framework (Earnshaw et al., 2013). This scale measured participants’ expectations of experiencing HIV-related stigma from family members, community, and healthcare providers. Sample items included “Family members will avoid touching me,” “Community managers will refuse to provide me with social services,” and “Healthcare providers will treat me with less respect.” Each item was rated on a scale of 1 (“definitely not”) to 5 (“definitely”), and a sum score was calculated (ranging from 9 to 45), with higher scores indicating higher levels of anticipated HIV stigma. The scale exhibited excellent internal consistency (Cronbach’s α = 0.93).

#### Enacted HIV-related stigma

Enacted HIV-related stigma was evaluated using a 16-item checklist adapted from a previous study (dos Santos et al., 2014). Participants indicated whether they had experienced specific instances of stigmatization due to HIV in the past 6 months, such as “Being excluded from social gatherings or activities,” “Being excluded from family activities,” and “Being physically assaulted.” Responses were dichotomous (1 = yes, 0 = no), and a composite score was calculated based on the total number of experienced stigmatizing events. The scale demonstrated good reliability (Cronbach’s alpha = 0.86).

### Statistical analysis

First, descriptive analysis was conducted to summarize participants’ sociodemographic characteristics using mean (standard deviation [SD]) for continuous variables and frequency (percentage [%]) for categorical variables.

Second, internal consistencies, means, standard deviations, skewness and kurtosis of the sum scores for each scale in each MSM group were examined. Normality of data distribution was assessed based on skewness (−2 to +2) and kurtosis (−7 to +7) criteria (Hair et al., 2010). Internal consistency above 0.7 was considered good and above 0.8 was considered great (Cortina, 1993).

Third, measurement invariance analysis was conducted following the procedure outlined by Vandenberg and Lance (2000). Multi-group comparisons in the context of CFA were performed using M*plus* 8.4 (Muthén and Muthen, 2017). The step-up approach was utilized to systematically introduce a series of increasingly rigorous equality constraints into the models (Brown, 2015). First, configural invariance of the baseline model was tested with multiple group comparisons wherein no equality constraints were imposed, to assess whether the same factor structure holds across groups. Second, metric invariance was examined by constraining factor loadings of indicators to be equal across groups. Third, scalar invariance was tested to determine whether item intercepts are equal, enabling meaningful group mean comparisons. For internalized and anticipated HIV-related stigma scale, characterized by continuous item responses, scalar invariance was tested by constraining intercepts of indicators to be equal across groups. In contrast, for the HIV-related enacted stigma scale, with dichotomous item responses, scalar invariance was evaluated by constraining item thresholds to be equivalent across groups. Item thresholds are specifically relevant for binary indicators (e.g., 0 = No, 1 = Yes), referring to the level of the latent trait (e.g., enacted stigma) that is associated with transitioning from being negative on the indicator to being positive on the indicator (Brown, 2015). Fourth, residual invariance was tested to determine whether item residual variances are equal, ensuring similar measurement precision across groups. For the internalized and anticipated HIV-related stigma scales, residual invariance was assessed by constraining item residual variances to be equal between groups. For the HIV-related enacted stigma scale, residual invariance was further evaluated by constraining the residual variances to 1 in both groups.

In cases where full measurement invariance could not be established, partial invariance was explored further (Byrne et al., 1989). By means of modification indices, a modified model for checking partial invariance by releasing the equality constraints for misspecified items was subsequently examined. To establish partial measurement invariance, at least the loadings/intercepts/thresholds/residuals of half of the scale items should be equal across groups (Byrne et al., 1989).

For the internalized and anticipated HIV-related stigma scales, involving continuous item responses, estimations were conducted with the *Mplus* maximum likelihood with robust standard errors (MLR) estimator, which adjusts the estimated standard errors for deviations from multivariate normality. For HIV-related enacted stigma scale, comprising dichotomous item responses, estimations were carried out using the *Mplus* weighted least squares mean and variance adjusted (WLSMV) estimator (Hansson and Gustafsson, 2013).

Following the recommendations of Hu and Bentler (1999), several fit indices including the root mean square error of approximation (RMSEA), standardized root mean square residual (SRMR), comparative fit index (CFI), and Tucker-Lewis Index (TLI) were employed to evaluate model fit, in addition to the Chi-square statistic., Thresholds for adequacy included RMSEA of 0.08 or less, SRMR of less than 0.08, and CFI and TLI of 0.95 or greater (Browne and Cudeck, 1992; Hu and Bentler, 1999; Weiber and Mühlhaus, 2014).

Model acceptance or rejection decisions were based on Chi-square difference tests (Hu and Bentler, 1999), where the Chi-square difference statistic was utilized across steps to determine if additional constraints significantly deteriorated fit (Bryant et al., 1997). For the internalized and anticipated HIV-related stigma scales, employing the MLR estimator, the ordinary Chi-square difference test was inappropriate, necessitating the use of the Satorra-Bentler scaled Chi-square difference test to obtain correct results (Satorra and Bentler, 2010). The test statistic, T, was calculated with the following equation, where c0 refers to scaling correction factor for the null model; c1 refers scaling correction factor for the alternative model; d0 refers to degrees of freedom for the null model; d1 refers to degrees of freedom for the alternative model; SB0 refers to Satorra-Bentler scaled Chi-square value for the null model; SB1 refers to Satorra-Bentler scaled Chi-square value for the alternative model. T is distributed Chi-square with degrees of freedom (d0 - d1) (Satorra and Bentler, 2010).


$$T=\frac{\left(SB0\times c0-SB1\times c1\right)\times \left(d0-d1\right)}{d0\times c0-d1\times c1}$$

For the HIV-related enacted stigma scales, utilizing the WLSMV estimator, Chi-square difference tests were conducted using the DIFFTEST option (Muthén and Muthen, 2017).

Scalar invariance is considered necessary and sufficient evidence for measurement invariance (Muthén and Muthen, 2017).

## Results

### Descriptive statistics

Table 1 summarizes the sociodemographic characteristics of non-MSM and MSM. Among the 1,174 MLWH, the majority were MSM (595, [50.7%]), aged between 35 and 44 years (454, [38.7%]), of Han ethnicity (766, [65.2%]), single (636, [54.2%]), with a middle school degree or below (479, [40.8%]), employed full-time (786, [67.0%]), with a monthly household income between 2,000 and 3,999 RMB (583, [49.7%]), a CD4 count less than 500 cells/mm3 (622, [53.0%]), and a viral load less than 50 copies/ml (1,022, [87.1%]).

**Table 1:** Sociodemographic characteristics by MSM status.

Variables	Overall (*N* = 1,177)	Non-MSM (*n* = 579)	MSM (*n* = 595)	*p*-value
Age group				**<0.001**
18–24	164 (14.0%)	13 (2.2%)	151 (25.4%)	
25–34	454 (38.7%)	139 (24.0%)	315 (52.9%)	
35–44	320 (27.3%)	228 (39.4%)	92 (15.5%)	
45+	235 (20.0%)	198 (34.2%)	37 (6.2%)	
Ethnicity				0.99
Han	766 (65.2%)	378 (65.3%)	388 (65.2%)	
Minority	408 (34.8%)	201 (34.7%)	207 (34.8%)	
Marital status				**<0.001**
Single	636 (54.2%)	134 (23.1%)	502 (84.4%)	
Married/life partner	404 (34.4%)	356 (61.5%)	48 (8.1%)	
Divorced/separated/widowed	134 (11.4%)	89 (15.4%)	45 (7.6%)	
Education				**<0.001**
Middle school and below	479 (40.8%)	393 (67.9%)	86 (14.5%)	
High school	259 (22.1%)	111 (19.2%)	148 (24.9%)	
College and above	435 (37.1%)	74 (12.8%)	361 (60.7%)	
Employment				**<0.001**
Fulltime	786 (67.0%)	374 (64.6%)	412 (69.2%)	
Parttime	191 (16.3%)	117 (20.2%)	74 (12.4%)	
Unemployed/retired	189 (16.1%)	81 (14.0%)	108 (18.2%)	
Monthly household income (RMB)				**<0.001**
<2,000	297 (25.3%)	191 (33.0%)	106 (17.8%)	
2,000-4,000	583 (49.7%)	298 (51.5%)	285 (47.9%)	
4,000 or above	294 (25.0%)	90 (15.5%)	204 (34.3%)	
CD4 count				**<0.001**
<500 cells/mm^3^	622 (53.0%)	369 (63.7%)	253 (42.5%)	
≥500 cells/mm^3^	552 (47.0%)	210 (36.3%)	342 (57.5%)	
Viral load				**0.02**
<50 copies/ml	1,022 (87.1%)	522 (90.2%)	500 (84.0%)	
≥50 copies/ml	140 (11.9%)	56 (9.7%)	84 (14.1%)	

Bivariate analyses between MSM status and sociodemographic characteristics were tested using chi-square test or Fisher’s exact test as appropriate. Bold indicates statistical significance.

Table 2 displays the internal consistencies, means, standard deviations, skewness, and kurtosis of the sum scores for each stigma scale within each group. Based on Hair et al. (2010) criteria (skewness≤2; kurtosis≤7), internalized stigma and anticipated stigma were normally distributed, while enacted stigma was not. The internal consistency was excellent for internalized stigma and anticipated stigma (*α* >0.8) and good for the enacted stigma scale (α >0.7).

**Table 2:** Internal consistencies, means, standard deviations, skewness and kurtosis of the sum scores for each HIV-related stigma scale in each MSM group.

Scale	Non-MSM					MSM				
	*M*	SD	Skew	Kurt	α	*M*	SD	Skew	Kurt	α
Internalized	16.14	5.49	0.36	0.20	0.94	17.32	6.01	0.10	−0.50	0.96
Anticipated	23.13	7.80	0.11	−0.28	0.93	23.41	7.92	0.12	−0.15	0.93
Enacted	0.95	2.15	3.64	−0.28	0.86	0.69	1.83	4.33	23.96	0.86

### Measurement invariance of internalized HIV-related stigma scale between MSM groups

Table 3 presents the results of multi-group tests of measurement invariance of the internalized HIV-related stigma scale. Model fit indices of the baseline model were indicative of configural measurement invariance (χ^2^ = 66.00, *df* = 31; CFI = 0.989; TLI = 0.980; RMSEA = 0.044, 95%CI [0.029, 0.059]; SRMR = 0.017).

**Table 3:** Summary of fit indices from invariance analyses between MSM groups for HIV-related internalized stigma scale.

Model	RMSEA (95% CI)	SRMR	CFI	TLI	χ^2^	*df*	*T*	Δ*df*	*p*-value^a^	Decision
Configural	0.044 (0.029, 0.059)	0.017	0.989	0.980	66.00	31				
Metric	0.043 (0.030, 0.056)	0.025	0.987	0.980	79.18	38	10.57	7	0.16	Accept
Scalar	0.048 (0.036, 0.060)	0.028	0.981	0.976	104.84	45	36.75	7	<0.001	Reject
Partial scalar (free I2 3 6)	0.043 (0.030, 0.056)	0.033	0.986	0.981	89.32	43	8.84	4	0.07	Accept
Partial residual (free I2 3 6)	0.041 (0.028, 0.053)	0.040	0.986	0.983	92.29	47	5.79	5	0.33	Accept

^ap-value indicates the significance of Satorra-Bentler scaled chi-square difference test.^

In the model imposing metric measurement invariance, item loadings were constrained to be equal between MSM groups. Model fit indices indicated satisfactory fit (χ^2^ = 79.18, *df* = 38; CFI = 0.987; TLI = 0.980; RMSEA = 0.043, 95%CI [0.030, 0.056]; SRMR = 0.025). A comparison of the metric model with the configural model using a Satorra-Bentler scaled chi-square difference test revealed no significant deterioration in fit (T = 10.57, Δ*df* = 7, *p* = 0.16), supporting metric invariance.

Scalar invariance was then examined by constraining intercepts of indicators to be equal between MSM groups. The fit of scalar measurement invariance was acceptable (χ^2^ = 104.84, *df* = 45; CFI = 0.981; TLI = 0.976; RMSEA = 0.048, 95%CI [0.036, 0.060]; SRMR = 0.028). However, comparison with the metric model showed significant deterioration in fit (T = 36.75, Δ*df* = 7, *p* < 0.001), indicating lack of scalar invariance. Partial scalar measurement invariance was established by allowing the intercepts of item 2, 3, and 6 to vary between groups (χ^2^ = 89.32, *df* = 43; CFI = 0.986; TLI = 0.981; RMSEA = 0.043, 95%CI [0.030, 0.056]; SRMR = 0.033), with no significant difference compared to the metric model (T = 8.84, Δ*df* = 4, *p* = 0.07).

Equivalence of the item residuals was constrained between MSM groups to examine residual invariance. Because the criteria were only met for partial scalar measurement invariance by allowing the intercepts of item 2, 3, and 6 to vary between groups, partial residual invariance was tested with residual item variances constrained to be equal between groups, except for the residual variance of item 2, 3, and 6. The fit of partial residual measurement invariance was good (χ^2^ = 92.29, *df* = 47; CFI = 0.986; TLI = 0.983; RMSEA = 0.041, 95%CI [0.028, 0.053]; SRMR = 0.040). Satorra-Bentler scaled chi-square difference test showed that the partial residual model was not significantly worse than the partial scalar model (T = 5.79, Δ*df* = 5, *p* = 0.33), supporting partial residual invariance across MSM groups.

### Measurement invariance of anticipated HIV-related stigma scale between MSM groups

The results of multi-group tests of measurement invariance of anticipated HIV-related stigma scale are presented in Table 4. Model fit indices of the baseline model of the scale were in line with configural measurement invariance (χ^2^ = 74.69, *df* = 46; CFI = 0.994; TLI = 0.990; RMSEA = 0.033, 95%CI [0.018, 0.046]; SRMR = 0.019).

**Table 4:** Summary of fit indices from invariance analyses between MSM groups for HIV-related anticipated stigma scale.

Model	RMSEA	SRMR	CFI	TLI	χ^2^	*df*	T	Δ*df*	*p*-value^a^	Decision
Configural	0.033 (0.018, 0.046)	0.019	0.994	0.990	74.69	46				
Metric	0.031 (0.017, 0.044)	0.021	0.993	0.991	82.00	52	5.17	6	0.52	Accept
Scalar	0.031 (0.017, 0.043)	0.021	0.993	0.991	90.27	58	7.41	6	0.28	Accept
Residual	0.034 (0.023, 0.045)	0.022	0.990	0.989	113.20	67	17.85	9	0.04	Reject
Partial residual (free I2)	0.027 (0.012, 0.038)	0.022	0.994	0.993	93.57	66	8.44	8	0.39	Accept

^ap-value indicates the significance of Satorra-Bentler scaled chi-square difference test.^

In the model imposing metric measurement invariance, item loadings were constrained to be equal between MSM groups. Model fit indices revealed satisfied fit (χ^2^ = 82.00, *df* = 52; CFI = 0.993; TLI = 0.991; RMSEA = 0.031, 95%CI [0.017, 0.043]; SRMR = 0.021). A comparison of the metric model with the configural model using a Satorra-Bentler scaled chi-square difference test showed that the more restrictive model with equal factor loadings was not significantly worse than the configural model (T = 5.17, Δ*df* = 6, *p* = 0.52), suggesting that the fit of metric invariance was satisfied.

To examine scalar invariance, intercepts of indicators were also constrained to be equal between MSM groups. The fit of scalar measurement invariance was good (χ^2^ = 90.27, *df* = 58; CFI = 0.993; TLI = 0.991; RMSEA = 0.031, 95%CI [0.017, 0.043]; SRMR = 0.021). A comparison of the scalar model with the metric model using a Satorra-Bentler scaled chi-square difference test showed that the scalar model was not significantly worse than the metric model (T = 7.41, Δ*df* = 6, *p* = 0.28), suggesting that the fit of scalar invariance was satisfied.

In addition to item slopes and intercepts, equivalence of the item residuals was constrained between MSM groups to examine residual invariance. The fit of residual measurement invariance was good (χ^2^ = 113.20, *df* = 67; CFI = 0.990; TLI = 0.989; RMSEA = 0.034, 95%CI [0.023, 0.045]; SRMR = 0.022). Satorra-Bentler scaled chi-square difference test showed that the partial residual model was significantly worse than the partial scalar model (T = 17.85, Δ*df* = 9, *p* = 0.04), suggesting that the fit of residual invariance was not satisfied. Therefore, residual of item 2 was allowed to vary between MSM groups to establish partial residual invariance (χ^2^ = 93.57, *df* = 66; CFI = 0.994; TLI = 0.993; RMSEA = 0.027, 95%CI [0.012, 0.038]; SRMR = 0.022). Satorra-Bentler scaled chi-square difference test showed that the partial residual model was not significantly worse than the scalar model (T = 8.44, Δ*df* = 8, *p* = 0.39), supporting partial residual invariance across MSM groups.

### Measurement invariance of enacted HIV-related stigma scale between MSM groups

The results of multi-group tests of measurement invariance of enacted HIV-related stigma scale are presented in Table 5. Model fit indices of the baseline model of the scale were in line with configural measurement invariance (χ^2^ = 312.24, *df* = 196; CFI = 0.984; TLI = 0.980; RMSEA = 0.032, 95%CI [0.025, 0.038]; SRMR = 0.087).

**Table 5:** Summary of fit indices from invariance analyses between MSM groups for HIV-related enacted stigma scale.

Model	RMSEA	SRMR	CFI	TLI	χ^2^	*df*	*p*-value^a^	Decision
Configural	0.032 (0.025, 0.038)	0.087	0.984	0.980	312.24	196		
Metric	0.028 (0.020, 0.034)	0.088	0.987	0.985	301.23	208	0.73	Accept
Scalar	0.028 (0.021, 0.034)	0.088	0.986	0.985	319.74	220	0.02	Reject
Partial scalar (free I7)	0.027 (0.020, 0.034)	0.088	0.987	0.986	314.74	219	0.16	Accept
Partial residual (free I7)	0.031 (0.024, 0.037)	0.087	0.985	0.982	316.41	204	0.55	Accept

^ap-value indicates the significance of chi-square value for ULSMV difference testing, which was done using the DIFFTEST option in Mplus 8.^

In the model imposing metric measurement invariance, item loadings were constrained to be equal between MSM groups. Model fit indices revealed satisfied fit (χ^2^ = 301.23, *df* = 208; CFI = 0.987; TLI = 0.985; RMSEA = 0.028, 95%CI [0.020, 0.034]; SRMR = 0.088). A comparison of the metric model with the configural model using chi-square value for ULSMV difference test showed that the more restrictive model with equal factor loadings was not significantly worse than the configural model (*p* = 0.73), suggesting that the fit of metric invariance was satisfied.

To examine scalar invariance, item thresholds were also constrained to be equal between MSM groups. The fit of scalar measurement invariance was good (χ^2^ = 319.74, *df* = 220; CFI = 0.986; TLI = 0.985; RMSEA = 0.028, 95%CI [0.021, 0.034]; SRMR = 0.088). A comparison of the metric model with the configural model using chi-square value for ULSMV difference test showed that the scalar model was significantly worse than the metric model (*p* = 0.02), suggesting that the fit of scalar invariance was not satisfied. Partial scalar measurement invariance was established by allowing the threshold of item 7 to vary between groups (χ^2^ = 314.74, *df* = 219; CFI = 0.987; TLI = 0.986; RMSEA = 0.027, 95%CI [0.020, 0.034]; SRMR = 0.088). Chi-square value for ULSMV difference test showed that the partial scalar model was not significantly worse than the metric model (*p* = 0.16), supporting partial scalar invariance across MSM groups.

Residual invariance was tested by constraining residual variance to 1 in both groups. Because the criteria were only met for partial scalar measurement invariance by allowing the threshold of item 7 to vary between groups, partial residual invariance was tested with residual variances constrained to be 1 in both groups, except for the residual variance of item 7. The fit of partial residual measurement invariance was good (χ^2^ = 316.41, *df* = 204; CFI = 0.985; TLI = 0.982; RMSEA = 0.031, 95%CI [0.024, 0.037]; SRMR = 0.087). Chi-square value for ULSMV difference test showed that the partial residual model was not significantly worse than the partial scalar model (*p* = 0.55), supporting partial residual invariance across MSM groups.

## Discussion

All four levels of measurement invariance, including configural, metric, scalar, and residual invariance, of the internalized, anticipated, and enacted HIV-related stigma scales were examined between 595 MSM and 579 non-MSM living with HIV. This study is a prerequisite for using these scales to assess internalized, anticipated, and enacted HIV-related stigma in between-group comparisons. The anticipated HIV-related stigma scale had the same factor loadings and intercepts, and similar item residual variances in the two groups and achieved partial residual invariance. The internalized and enacted HIV-related stigma scales had the same factor loadings and similar intercepts in the two groups and achieved partial scalar invariance. Scalar invariance is considered the minimum requirement for meaningfully comparing latent factor means across groups (Muthén and Muthen, 2017). This study suggested that measurement invariance of all three HIV-related stigma scales was satisfied.

The multi-group tests of measurement invariance of internalized HIV-related stigma scale suggested that both configural and metric invariances were fully satisfied between MSM and non-MSM, indicating that the scale intervals are the same across groups, allowing for comparing unstandardized regression coefficients and/or covariances across groups (Pirralha, 2020). The scalar invariance was only partially satisfied, by freeing the constraint of intercepts of item 2 (“I feel ashamed of having HIV”), item 3 (“Having HIV makes me feel unclean”), and item 6 (“I feel guilty because I have HIV”) across groups. These results indicated that the zero points of these items were different between MSM and non-MSM. That is, MSM were more likely to feel “ashamed,” “unclean,” and “guilty,” but the increased levels of these feelings were not related to increased levels of internalized HIV-related stigma among MSM. In line with a qualitative study describing stigma related to pre-exposure prophylaxis (PrEP) uptake among HIV-negative MSM, participants reported that they were already losing respect, and they felt guilty and ashamed using PrEP due to their MSM identities, while their straight friends were not ashamed to talk about HIV (Dubov et al., 2018). MSM often face social disapproval of sexual deviance from the “normal” sexual identity, producing the feeling of shame (Fortenberry et al., 2002; Herek, 2004). Even through such feeling of shame does not resulting from HIV, it could predict risky sexual behavior (e.g., unprotected sex), which elevates the risk of acquiring HIV (Newcomb and Mustanski, 2011).

The measurement invariance analysis of anticipated HIV-related stigma scale between MSM and non-MSM showed that the first three levels of measurement invariance including configural, metric, and scalar invariances were satisfied. Residual invariance was partially satisfied by freeing the constraint of residual variance of item 2 (“Family members will look down on me”) across groups. This result indicated that the item variance that could not be explained by the factor was different between MSM and non-MSM. For the MSM group, besides HIV-related stigma, the fear of negative responses from family members might also be explained by their MSM identities. A qualitative study about HIV disclosure reported that only 57.1% of MSM (vs. 72.2% of straight MLWH) disclosed their HIV seropositive status to family members (Ko et al., 2007). Compared to non-MSM, MSM had more concern that they did not want to explain to their family how they got this disease (Ko et al., 2007). Another qualitative study among MSM about disclosing their sexual identities to family members reported that responses from family members could be supportive, denial, confused, or unsupportive (Gyamerah et al., 2019). Whether or not family was supportive, silence around the MSM’s sexual identity was prevalent within families (Gyamerah et al., 2019).

Similar to internalized HIV-related stigma, the multi-group tests of measurement invariance of enacted HIV-related stigma scale suggested that both configural and metric invariances were satisfied between MSM and non-MSM. The scalar invariance was partially satisfied by freeing the constraint of intercept of item 7 (“stress from spouse/partner”) between two groups. This result indicated that threshold of item 7 was different between MSM and non-MSM. That is, MSM were more likely to experience “stress from spouse/partner” but this was not related to increased levels of enacted HIV-related stigma among MSM. For MSM, internalized homophobia and homophobic discrimination were both established to have significant associations with sexual partner violence (Finneran and Stephenson, 2014). Although Chinese societies became relatively tolerant toward MSM since homosexuality was removed from the Chinese Classification and Diagnostic Criteria of Mental Disorders in 2001 (Wu, 2003), same-sex marriage is still illegal, and discrimination based on sexual and/or gender identity are not prohibited by laws (Zhang and Chu, 2005; Cao and Guo, 2016). MSM are still expected to fulfill their family duty of heterosexual marriage and having children (Wu, 2003). Such stress from spouse or partner could further lead to psychological distress and stress-sensitive illness (Sun et al., 2020).

From methodological perspectives, study results indicated that the three HIV-related stigma scales were all acceptable for use in between-group comparisons. Previous studies have validated the internal consistency and factor structure of the internalized HIV-related stigma scale among MSM living with HIV (Valle et al., 2015) and its short version in adolescents living with HIV (Wanjala et al., 2021). This study further provide evidence for the generalizability of this scale by directly comparing the measurement structure between MSM and non-MSM. Although the current study reported satisfied measurement invariance for the anticipated HIV-related stigma scale between MSM and non-MSM, the reliability and validity of this scale were controversial (Reinius et al., 2018; Brown et al., 2021). Reinius et al. (2018) suggested that the scale should be revised when a very high proportion of PLWH were under efficient treatment. Future studies are needed to confirm these findings and provide evidence for the valid use of anticipated HIV-related stigma scale. Similarly, although the enacted HIV-related stigma index has been used in 61 countries worldwide (Global Network of People Living with HIV, 2022) and has been used among MSM and female sex workers (Gottert et al., 2019; Gottert et al., 2020; Yam et al., 2020; Lo Hog Tian et al., 2021), limited studies provided evidence on its reliability, validity, and measurement invariance across groups of this scale. This study made new contributions to the measurement invariance of the enacted HIV-related stigma scale, supported its use in comparison studies between MSM and non-MSM.

This study is one of the very few studies comprehensively examining the measurement invariance of internalized, anticipated, and enacted HIV-related stigma between MSM and non-MSM. All four levels of measurement invariance (i.e., configural, metric, scalar, residual) were tested compared to previous studies that only tested the first three levels of measurement invariance (Miller and Sheu, 2008). The current study provides some insights for future research and interventions aimed at reducing HIV-related stigma across diverse groups. Prior research has demonstrated that multi-level stigma reduction interventions incorporating community engagement, policy advocacy, and psychosocial support have been effective in mitigating stigma and improving health outcomes for people living with HIV (Andersson et al., 2020). Also, education and structural interventions are essential in addressing HIV stigma, particularly when tailored to the specific needs of different populations (Feyissa et al., 2018; Daniel et al., 2019). These approaches align with our findings, suggesting that MSM-specific stigma interventions should integrate both HIV-related and sexual identity-related stigma reduction strategies to ensure effectiveness. Future research should explore how these interventions can be further adapted across cultural and social contexts to maximize their impact.

Several limitations should be noted. Firstly, our sample was drawn from MSM and non-MSM populations exclusively in China. Cultural norms, healthcare access, and societal attitudes toward HIV and MSM individuals vary across different regions, potentially influencing stigma experiences. As such, caution should be exercised when applying these findings to populations outside China. Future studies should aim to validate the measurement invariance of these stigma scales in other cultural contexts to enhance cross-national applicability. Second, differences in sociodemographic characters between MSM and non-MSM existed. MSM were more likely to be younger, single, have a college degree or above, fulltime employed with higher household income. Therefore, the result should be interpreted with caution. Future studies may consider propensity score matching when comparing samples possessing different characteristics. Third, self-reported measures of stigma might introduce recall bias and social desirability. However, for internalized and anticipated stigma, self-reporting is the most valid method, as these constructs inherently reflect an individual’s personal perceptions, which cannot be objectively measured. For enacted stigma, recall bias may be a concern, as participants may misremember or underreport past discriminatory experiences. To mitigate this, we used a six-month recall period, which helps balance capturing relevant experiences while reducing potential recall distortions. Future studies could complement self-reported measures with qualitative interviews or longitudinal assessments to provide deeper insights into stigma experiences over time. Fourth, participants were all recruited through the CDC health record system, whose health status could be well-maintained and better than the general PLWH population.

## Conclusion

Overall, this study presented acceptable measurement invariance for internalized, anticipated, and enacted HIV-related stigma scales between MSM and non-MSM. The invariance across groups should be interpreted with caution since the constraints of some items varied across groups. This study provided evidence and support for future studies using these scales to assess HIV-related stigma between MSM and non-MSM, which could be a basis for future intervention of stigma reduction.

## Data Availability

The original contributions presented in the study are included in the article/Supplementary material, further inquiries can be directed to the corresponding author.
