# Supplementary material for: Measurement invariance of HIV-related stigma scales among men who have sex with men (MSM) and non-MSM populations: implications for comparative studies in China
Source: Front Psychol. 2025 Apr 25;16:1510034. doi: 10.3389/fpsyg.2025.1510034 (PMC12061874; doi:10.3389/fpsyg.2025.1510034)
Supplement: Supplementary file 1 [file Table_1.docx]

Table A1. Internalized HIV-related stigma scale

| Rate each of the statements below using the following scale | | Strongly disagree | Disagree | Agree | Strongly agree |
| --- | --- | --- | --- | --- | --- |
| 1 | I feel guilty because I have HIV |  |  |  |  |
| 2 | I feel ashamed of having HIV |  |  |  |  |
| 3 | Having HIV makes me feel unclean |  |  |  |  |
| 4 | I feel I am not as good a person as others because I have HIV |  |  |  |  |
| 5 | I think less of myself because I have HIV |  |  |  |  |
| 6 | I feel guilty because I have HIV |  |  |  |  |
| 7 | Having HIV in my body is disgusting to me |  |  |  |  |
| 8 | People's attitudes about HIV make me feel worse about myself |  |  |  |  |
