# Supplementary material for: Measurement invariance of HIV-related stigma scales among men who have sex with men (MSM) and non-MSM populations: implications for comparative studies in China
Source: Front Psychol. 2025 Apr 25;16:1510034. doi: 10.3389/fpsyg.2025.1510034 (PMC12061874; doi:10.3389/fpsyg.2025.1510034)
Supplement: Supplementary file 2 [file Table_2.docx]

Table A2. Anticipated HIV-related stigma scale

| How likely is it that people will treat you in the following ways in the future because of your HIV status? | | Very unlikely | Unlikely | Neither unlikely nor likely | Likely | Very Likely |
| --- | --- | --- | --- | --- | --- | --- |
| 1 | Family members will avoid me |  |  |  |  |  |
| 2 | Family members will look down on me |  |  |  |  |  |
| 3 | Family members will treat me differently |  |  |  |  |  |
| 4 | Community/social workers won’t take my needs seriously |  |  |  |  |  |
| 5 | Community/social workers will discriminate against me |  |  |  |  |  |
| 6 | Community/social workers will deny me services |  |  |  |  |  |
| 7 | Healthcare workers will not listen to my concerns |  |  |  |  |  |
| 8 | Healthcare workers will avoid touching me |  |  |  |  |  |
| 9 | Healthcare workers will treat me with less respect |  |  |  |  |  |
