# Supplementary material for: Measurement invariance of HIV-related stigma scales among men who have sex with men (MSM) and non-MSM populations: implications for comparative studies in China
Source: Front Psychol. 2025 Apr 25;16:1510034. doi: 10.3389/fpsyg.2025.1510034 (PMC12061874; doi:10.3389/fpsyg.2025.1510034)
Supplement: Supplementary file 3 [file Table_3.docx]

Table A3. Enacted HIV-related stigma scale

| Have people treated you this way in the past 6 months because of your HIV status? | | Yes | No |
| --- | --- | --- | --- |
| 1 | Excluded from social gatherings |  |  |
| 2 | Excluded from family activities |  |  |
| 3 | Being gossiped about |  |  |
| 4 | Verbally insulted/harassed, threatened |  |  |
| 5 | Physically harassed or threatened |  |  |
| 6 | Physically assaulted |  |  |
| 7 | Husband/spouse/other household member have been discriminated against |  |  |
| 8 | Sexual rejection |  |  |
| 9 | Discriminated against by other PLWH |  |  |
| 10 | Other household members being discriminated because I have HIV |  |  |
| 11 | Forced to move or rejected for house leasing |  |  |
| 12 | Lose job or source of income |  |  |
| 13 | Rejected by employer or being fired |  |  |
| 14 | Forced to change job or denied promotion |  |  |
| 15 | Children being rejected by educational institutions because I have HIV |  |  |
| 16 | Denied by healthcare facilities |  |  |
